# Dynamic Physician Rostering in Emergency Departments: Managing Reentrant Patient Flows to Meet Excess Wait Time Targets

**DOI:** 10.1002/hcs2.70100

**Published:** 2026-09-11

**Authors:** Xu Dai, Peng Gao, Xiaolei Xie

**Affiliations:** ^1^ Department of Industrial Engineering Tsinghua University Beijing China; ^2^ Department of Medical Affairs China‐Japan Friendship Hospital Beijing China

**Keywords:** emergency department, fluctuating demand, physician rostering, quality of care, reentrant patient, workload

## Abstract

**Background:**

This study optimizes physician rostering for emergency departments facing fluctuating patient volumes and complex flow dynamics. Specifically, we address the challenge of patient reentry, including those who leave without being seen and subsequently return (pre‐service returns), as well as patients leaving subsequent to being seen requiring repeat evaluation or treatment for unresolved conditions (post‐service revisits). These endogenous patient flows significantly disturb the clinical workload and complicate capacity planning.

**Methods:**

We develop a patient flow model that integrates these return loops and formulate a risk‐aware physician rostering framework designed to strictly enforce excess wait time targets, for example, tail probability of delay service levels. We employ a simulation‐based iterative staffing algorithm to efficiently compute the optimal physician rules required hour‐by‐hour.

**Results:**

Discrete‐event simulation experiments demonstrate that this approach effectively aligns workload with demand to maintain care standards without incurring unnecessary labor costs.

**Conclusions:**

This study develops a simulation‐based, risk‐aware physician rostering framework for emergency departments with reentrant patient flows to meet excess wait time targets such as tail probability of delay while reducing unnecessary workload pressure.

## Graphical abstract

We develop a patient flow model that integrates return loops and formulate a risk‐aware physician rostering framework designed to strictly enforce excess wait time targets, for example, tail probability of delay (TPoD) service levels. We employ a simulation‐based iterative staffing approach (ISA) to determine the optimal number of physicians required hour‐by‐hour.

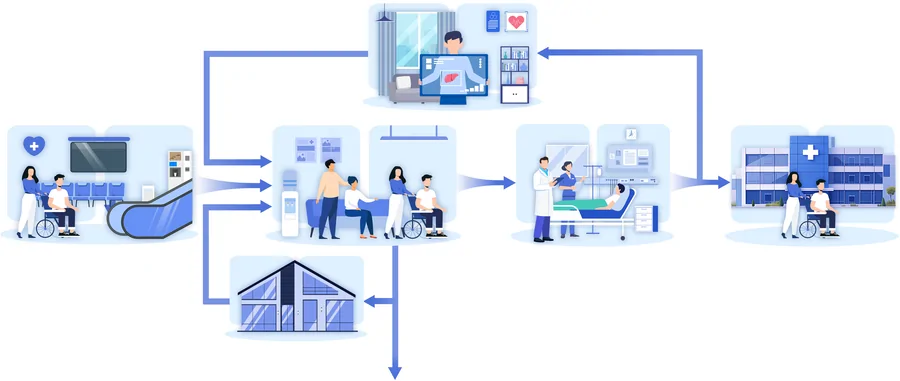

**Abbreviations**
ED: emergency department
Exp: experiment.
ISA: iterative staffing algorithm
LSBS: leave subsequent to being seen
LWBS: leave without being seen
SLA: service level agreement
TPoD: tail probability of delay

## 1 Background

Overcrowding and prolonged waiting times act as critical bottlenecks in emergency department (ED) operations worldwide. While patient arrivals follow strong diurnal patterns, clinical capacity (staff, beds) is often rigid. This mismatch leads to access block, compromising patient safety and satisfaction. Operationally, excessive waiting drives up patients' leaving without being seen (LWBS) rates. Crucially, clinical data shows a significant portion of those patients return to the ED within 1–3 days, often with worsened conditions. Similarly, patients left with unresolved issues may re‐enter the care process. These reentrant flows create a hidden demand load that traditional staffing models often overlook, leading to mis‐staffing and persistent failure to meet service level agreements (SLAs). In practice, such mismatch not only harms access but also increases clinicians' workload intensity and overtime risk.

Patients who leave before treatment is complete commonly include LWBS, defined as departure before completing the medical screening exam, and left subsequent to being seen (LSBS), defined as departure after medical screening exam completion but in fact before disposition. Empirical evidence underscores the scale of this issue. For instance, in a study of 626,548 ED visits, Smalley et al. identified 20,158 leave before treatment complete index encounters (3.2%), among which 6745 (33.5%) returned within 30 days [1]. Notably, 41.7% of these returned within 1 day; 76.1% returned within 10 days, and 66.4% returned to the selected ED. The median time to return was 43.3 h, and one fifth of returning patients were subsequently admitted. Vilke et al. reported that, over a 7‐month period with 50,766 ED visits, 1847 patients (3.6%) had LWBS [2]. Among these LWBS patients, 308 (16.7%) returned to one of the EDs within 1 day for examination; the cumulative return rate increased to one‐fifth within 2 days, 22.3% within 3 days, and 27.1% within 7 days.

In healthcare delivery and other service settings, repeat demand typically arises through three mechanisms, which ED physicians and managers have not yet incorporated into practice.

(1) Post‐service feedback, i.e., revisit after LSBS [3, 4, 5]: Patients who require additional care after a completed visit return to the same ED. Under a Bernoulli feedback structure with random revisit delays, Liu and Whitt further developed DIS‐OL and DIS‐MOL staffing rules to stabilize the expected delay and the abandonment probability [3]. For nonstationary feedback queues, Yom‐Tov and Mandelbaum adjusted the workload and derived a fluid approximation to stabilize the delay probability [5].

(2) Pre‐service retrial, that is, return after LWBS [6, 7, 8, 9]: Patients may leave before being seen because of limited patience or perceived congestion, and then attempt to seek care again after some time. Along this line, Wang et al. put forward a joint service‐and‐retrial discretization rule that mitigates delay without altering the overall service capacity [9].

(3) The combination of post‐service feedback and pre‐service retrial [10, 11]: In such networks, patients may both leave‐and‐return and also re‐enter after completing a prior visit. In this setting, Furman et al. studied time‐varying arrivals with abandonment, pre‐service retrial, and post‐service feedback in a multi‐class queueing system and proposed new fluid approximations for service‐quality control [12]. These returning behaviors are prevalent in ED operations; they make patient demand harder to characterize and, in turn, complicate physician capacity planning and rostering. Moreover, Defraeye and Van Nieuwenhuyse highlighted that systems with joint retrial and feedback dynamics warrant further investigation [13].

Many care delivery systems operate under fluctuating demand, in the sense that patient arrivals (visits) vary stochastically over time. In this context, capacity planning is a key lever for maintaining access and service quality; Defraeye and Van Nieuwenhuyse provided an overall literature review and emphasized the central role of resource planning decisions in diurnal patterns and complex environments [13]. When the underlying queueing model becomes too intricate for tractable analysis, simulation‐driven approaches offer a practical alternative for service level control. In particular, Feldman et al. introduced an iterative, simulation‐based iterative staffing algorithm (ISA) approach for delay probability stabilization in large nonstationary queues [14]. Then, the authors refined this idea into an exploration‐exploitation procedure to stabilize targeted performance and search for effective time‐dependent shift capacity [15, 16].

Beyond these simulation‐based methods, recent work has also pursued learning‐based and data‐driven paradigms to handle intractable fluctuating demand. Dai et al. not only built an Actor‐Critic neural network architecture for queueing network control, but also proposed an approximate martingale process method to reduce the variance of policy gradient estimation [17]. It has been proved that under various conditions ranging from light load to heavy load, the dynamic scheduling and resource routing strategies generated by deep reinforcement learning are superior to the existing state‐of‐the‐art heuristic algorithms in the literature. Chen et al. proposed an online learning framework designed for solving a dynamic pricing and capacity sizing problem in a GI/GI/1 queue that does not require the system's scale to increase [18]. Chen et al. proposed an online gradient descent solution mechanism with real data feedback, which proves that the optimal control of the steady‐state performance of complex queueing systems can still be achieved without assuming the underlying distribution law [19]. Baron et al. proposed a fully data‐driven framework that utilizes machine learning to identify variable parent sets and causal topological relationships in queueing systems, which can accurately reproduce the data‐generating process of a real queueing system without prior knowledge of queueing dynamics [20].

The ED care process can be modeled as a nonstationary care delivery system with fluctuating external arrivals, together with clinical re‐entries or return visits. Accordingly, this paper develops a modeling and simulation framework to support physician capacity planning while meeting quality of care targets, such as the expected delay and the tail probability of delay. Building on the above literature, we aim to explicitly incorporate these reentrant behaviors into the capacity analysis. Specifically, we construct a patient flow model that captures the dynamics of the ED service process and use a simulation‐based ISA approach to guide efficient, time‐dependent physician rostering. The main contributions of this study are as follows:

(1) (Modeling) We formulate a time‐dependent reentrant patient flow model for ED operations, in which return after LWBS and revisit after LSBS flows are jointly incorporated to capture endogenous reentrant demand, which will significantly disturb the arrival characteristics of patient flow.

(2) (Modeling) We adopt the tail probability of delay (TPoD) as our primary quality‐of‐care metric. While ED managers have traditionally focused on average waiting times, TPoD provides a more rigorous risk‐based standard that has yet to be widely incorporated into practice.

(3) (Algorithm) We apply an ISA approach to compute time‐dependent physician rostering rules that target a prescribed TPoD, and we demonstrate its effectiveness via discrete‐event simulation.

A distinctive feature of EDs is that patient demand is highly fluctuating and largely unscheduled. Patients arrive through walk‐ins and ambulance transports, undergo triage upon arrival, and then join different care pathways depending on their acuity level.

### 1.1 The Patient Flow

Limited physician capacity, treatment rooms, and diagnostic resources (e.g., imaging and laboratory services) often create patient backlog, leading to prolonged waiting and, in some cases, patients leaving before being seen. Meanwhile, some patients may return after an initial departure (retrial) or revisit due to relapse or unresolved conditions (feedback), which generates additional clinical reentries beyond the original demand.

**Figure 1:**
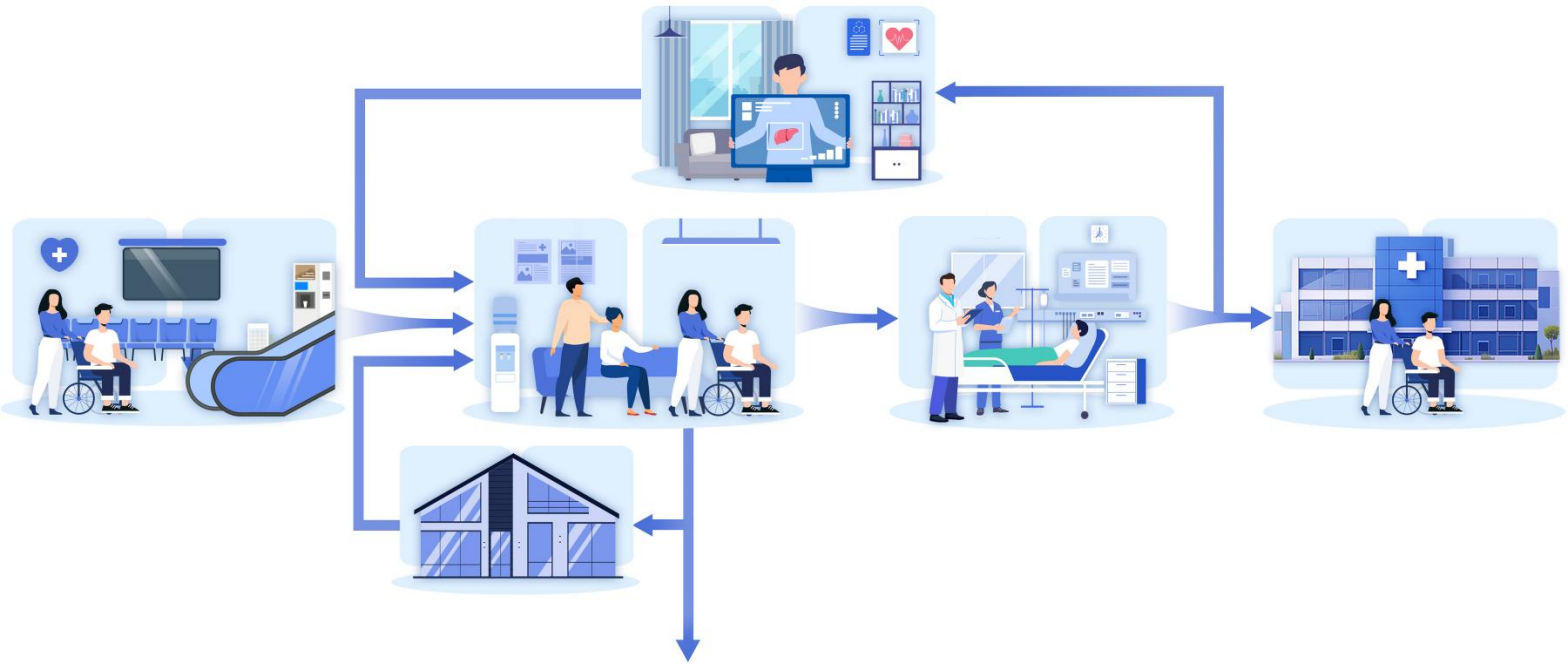
Flow diagram of the emergency department care delivery system. This figure was created by the authors.

Figure 1 summarizes the patient flow in an ED. Patients arrive exogenously, may wait in queue before treatment, and can abandon if their waiting exceeds their patience; abandoned patients may later retry and rejoin the system. After completing service, a fraction of patients may re‐enter the ED due to relapse or follow‐up needs, which induces a reentrant arrival stream in addition to the original demand.

### 1.2 Conceptual Model

In the meantime, ED operations exhibit complex patient behaviors, so both arrival and departure processes should be carefully represented. In particular, a portion of low‐acuity patients may leave the ED before being seen when faced with prolonged waits, and some of them will return later when symptoms persist or worsen (pre‐service retrial). Moreover, even after receiving treatment, a subset of patients may revisit the ED within a short time window due to relapse, complications, or unresolved conditions (post‐service feedback). To capture these ED‐specific dynamics in a tractable manner, we develop a notional model that abstracts the ED care procedure as a time‐dependent reentrant patient flow network with both return after LWBS and revisit after LSBS loops.

**Figure 2:**
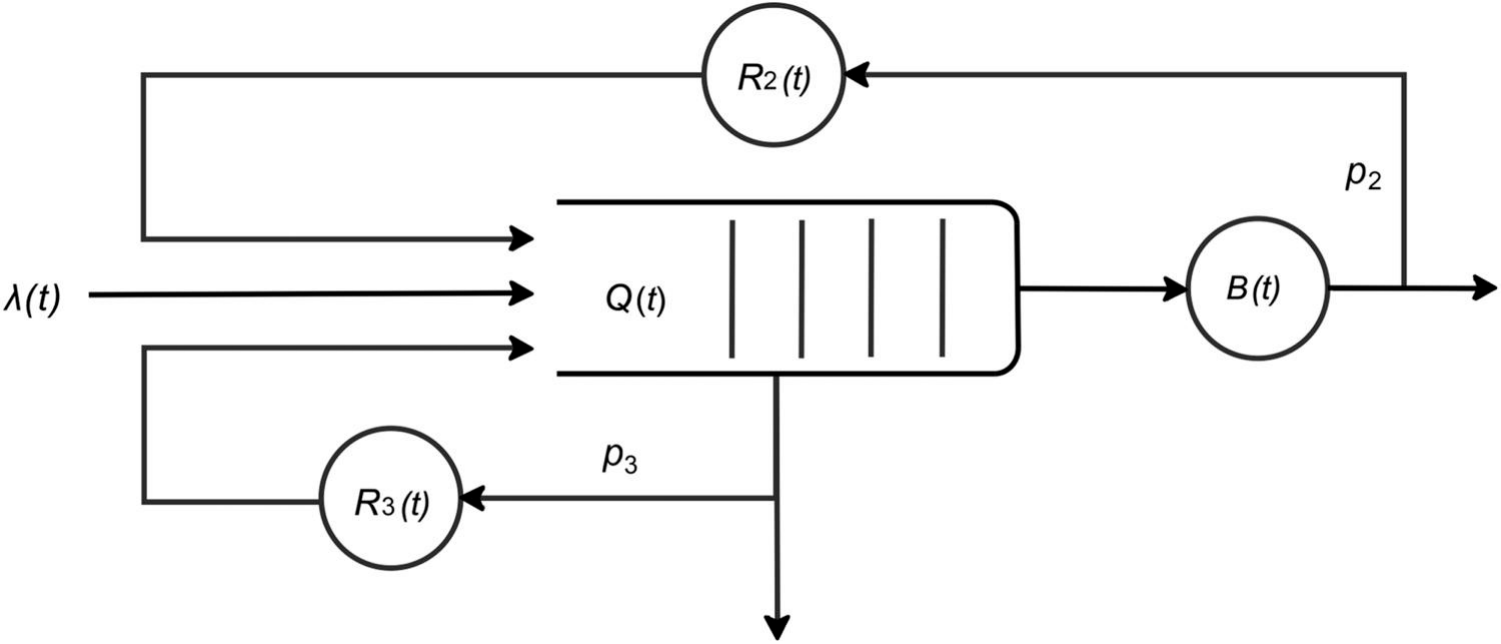
Patient flow with return after LWBS and revisit after LSBS. The ED is represented as a treatment area with B(t) parallel physicians and a single waiting room Q(t). (1) Pre‐service retrials, i.e., return after LWBS, where patients who leave without being seen join an “orbit” $R_{3}(t)$ and, after a random delay, attempt to seek care again (retrial flow) with probability $p_{3}$, and (2) post‐service feedback, that is, revisit after LSBS, where, after completing service, a fraction $p_{2}$ of patients in the “orbit” $R_{2}(t)$ revisit the waiting room after a random delay (feedback flow). ED, emergency department; LSBS, leave subsequent to being seen; LWBS, leave without being seen.

Figure 2 presents the patient flow abstraction of the care delivery process. The ED is represented as a treatment area with B(t) parallel physicians and a single waiting room Q(t); external arrivals feed the waiting room, and patients that cannot be seen immediately contribute to a fluctuating clinical workload. Two reentrant mechanisms are explicitly represented: (1) pre‐service retrials, i.e., return after LWBS, where patients who leave without being seen join an “orbit” $R_{3}(t)$ and, after a random delay, attempt to seek care again (retrial flow) with probability $p_{3}$, and (2) post‐service feedback, i.e., revisit after LSBS, where after completing service a fraction $p_{2}$ of patients in the “orbit” $R_{2}(t)$ revisit the waiting room after a random delay (feedback flow). This representation clarifies how revisit and return behaviors endogenously disturb patient demand and motivates physician rostering rules that target patient‐centered performance measures such as tail delay and abandonment.

## 2 Methods

To guide ED physician rostering rules under reentrant patient flows, we develop a simulation‐based approach that aims to stabilize system performance, that is, quality of care, over a planning horizon.

### 2.1 Performance Evaluation

To evaluate system performance via simulation, we introduce virtual patients and record key measures, including the excess delay probability (i.e., TPoD) and the expected delay. The time spacing between consecutive virtual patients is set to balance computational effort and estimation accuracy.

The interval $\left[t_{s},t_{f}\right)$ denotes the adjustment period during which the physician rostering level is updated. Over the planning horizon T, we introduce a time lag τ (i.e., the clinically meaningful delay threshold w) to account for indicator volatility.

The excess delay probability (i.e., TPoD), measures the probability that a patient arriving at time *t* experiences a waiting time longer than a prescribed threshold τ, that is, Pr{W(t)>τ}≤α for t∈[0,T]. Given a target level α, the physician rostering level aims to keep the time‐dependent waiting‐time distribution under control so that exceedances beyond τ occur with probability at most α. In the subsequent ISA‐based procedure, performance is evaluated over the shifted interval $\left[t_{s}-\tau ,t_{f}-\tau \right)$ to account for the lag between capacity changes and their effect on waiting‐time outcomes.

Managerial note (the “TPoD” metric): unlike manufacturing or service systems that often optimize for average throughput, EDs should prioritize patient safety by minimizing extreme delays. Meanwhile, ED physicians and managers have not yet incorporated TPoD into practice and instead focus primarily on the average waiting time metric. We therefore focus on the TPoD—effectively the “SLA breach rate”. This metric tracks the percentage of patients whose wait times exceed a clinically defined safety threshold (e.g., 30 min), offering a more rigorous standard for operational safety than simple averages.

### 2.2 Two‐Step ISA Framework

For the original ED setting with potentially repeated patient reentries, as illustrated by the patient flow structure in Figure 2, exact analysis becomes intractable. Fortunately, by using the simulation‐based ISA approach, we can efficiently search for time‐dependent physician rostering levels that meet the prescribed SLA target.

We implement a two‐step procedure. Step 1 involves exploration, that is, Supporting Information S1: Material 1: Algorithm 1, which rapidly identifies a physician rostering profile whose measure is near the targeted value, although it may not always be feasible. Step 2 focuses on exploitation (Supporting Information S2: Material 2: Algorithm 2) and leverages the exploration outcomes to further update the best‐known feasible rule yet. Here we amplify and update the physician rostering level for each time interval [*t*_s_, *t*_f_), $m_{j}(\tilde{t})=\;1+(\Phi_{\max,j}(\tilde{t})-\alpha )/\alpha j$, for $0\leq \tilde{t}\leq \;T$ where $m_{j}(\tilde{t})$ is the scale indicator, which is calculated according to the bias between the transient metric $\Phi_{\max,j}(\tilde{t})$ and the service level target α.

To provide a clearer operational intuition behind the proposed ISA, we detail the step‐by‐step logic of both the exploration and exploitation phases. Rather than relying on static formulas, the ISA dynamically “learns” the true demand by observing simulated patient flows, including the complex re‐entries. The primary goal of the exploration phase (Algorithm 1) is to rapidly identify a “safe” baseline staffing level that absorbs the hidden demand from reentrant patients and satisfies the TPoD safety threshold (*α*).

Step 1: Initialization. The algorithm begins with a deterministic baseline capacity, $B_{0}(t)=\;\left\lceil \lambda /\mu \right\rceil$. This represents the absolute minimum number of physicians required if patients flowed perfectly without diurnal variations or re‐entries.

Step 2: Simulation and risk assessment. We simulate the ED operations using the current staffing roster $B_{j}(\tilde{t})$ and observe the maximum TPoD, denoted as $\Phi_{\max,j}$. This step essentially asks: “Under the current schedule, what is the highest risk of patients experiencing unsafe wait times?”

Step 3: Dynamic capacity adjustment. If the observed delay risk exceeds the target $\Phi_{\max,j}(\tilde{t})>\;\alpha$, the scaling indicator $m_{j}(\tilde{t})$ becomes greater than 1, triggering an aggressive upward adjustment in physician capacity. Crucially, the adjustment step size is inversely proportional to the iteration count (*j*). This ensures rapid capacity expansion in the early rounds to clear severe bottlenecks, while preventing massive overshooting in later rounds.

Step 4: Convergence check. The loop terminates either when the staffing levels repeat (indicating the required capacity has been found) or when the stability indicator demonstrates that adding more physicians yields diminishing returns on service level improvements.

Because the exploration phase prioritizes patient safety and rapid convergence, it may slightly overshoot, resulting in localized overstaffing. The exploitation phase acts as a precision trimming tool to maximize labor efficiency without compromising the newly secured access standards.

Step 1: Baseline adoption. Algorithm 2 takes the safe‐but‐conservative staffing roster from Algorithm 1 as its starting point.

Step 2: Marginal reduction test. The algorithm identifies specific time intervals where the TPoD is significantly below the safety threshold *α* (i.e., hours where the ED is over‐performing). It tentatively reduces the physician headcount by exactly one resource $B(\tilde{t})-\;1$ in those targeted hours.

Step 3: Safety verification. A new simulation is triggered to test this leaner roster. If the ED still strictly meets the TPoD SLA (≤α), the reduction is locked in, effectively saving staffing costs.

Step 4: Reversion and termination. If removing a physician causes a localized SLA breach, the system immediately reverts that specific hour to its previous staffing level and marks it as “irreducible.” The algorithm terminates when no further reductions can be made across any time interval without violating the safety threshold, outputting the globally optimal rostering plan.

During the exploration of the physician rostering plan, it may fail to meet the service level target. Then we use exploitation to update the previous results and get an optimal physician rostering rule in step two. To monitor the convergence of our iterative approach, we introduce the squared coefficient of variation as a stability indicator. Let $c_{j}$ denote the cost associated with physician rostering level $B_{j}$; $c^{*}$ indicates the lowest cost of the feasible physician rostering plan up to now; $e_{j}$ represents the count of physician adjustments for which $\Phi_{\max,j}\;>\;\alpha$, with physician rostering level $B_{j}$; *u* means the unit physician cost for one physician‐adjustment (in physician hours). The default value of $c^{*}$ is set as the best cost of the feasible rule found in exploration step (if any); otherwise, it is set to be ∞.

With the two‐step ISA approach, we get an optimal time‐dependent physician rostering plan for the ED, which minimizes physician rostering cost while satisfying the service level target.

## 3 Results

We conduct experiments on a laptop running macOS Monterey (version 12.7, Apple Inc., Cupertino, United States). The hardware consists of an Intel Core i5 processor (2.9 GHz, Intel Corporation, Santa Clara, United States) with 16 GB of memory. We implement all the codes in MATLAB (MathWorks, Natick, United States), running simulations in the R2023b environment. This experimental design allows us to quantify how reentrant demand amplification affects performance and to verify that ISA can stabilize tail‐delay service levels without excessive overstaffing. Each simulation is conducted with 1000 replications.

**Figure 3:**
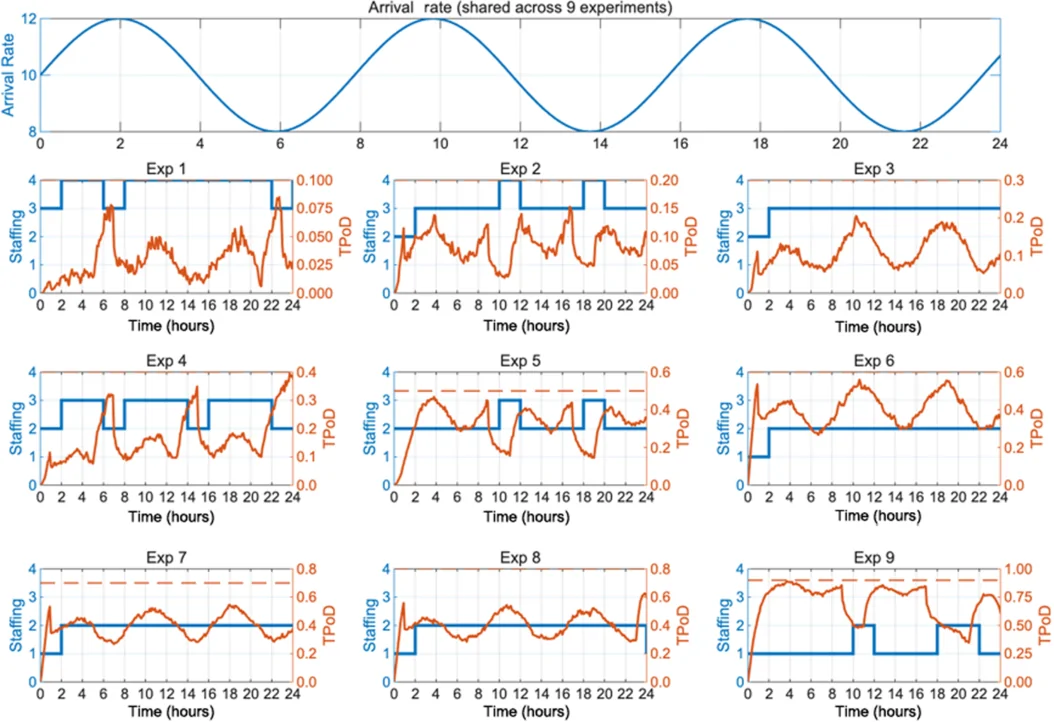
Dynamic alignment of physician capacity with patient demand over a 24‐h cycle with post‐service feedback probability *p*_2_ = 0.7 and pre‐service retrial probability *p*_3_ = 0.1. The horizontal *x*‐axis represents the time of day in hours. The vertical *y*‐axis illustrates three key operational metrics: (1) the fluctuating patient arrival rate (measured in patients per hour), (2) the recommended physician staffing level (expressed as the number of active physicians), and (3) the resulting TPoD value, indicating the probability of patients experiencing wait times that exceed the clinical safety threshold. Exps 1–9 respectively represent the TPoD ranging from 0.1 to 0.9. Abbreviations: Exp, experiment; TPoD, tail probability of delay.

Figure 3 shows the simulation outputs for the ED system under the reentrant setting, where $p_{2}=0.7$ is the post‐service feedback probability and $p_{3}=0.1$ is the pre‐service retrial probability. The subplots jointly illustrate how the ISA‐driven time‐dependent physician rostering plan adjusts system capacity over time and how the induced quality of care (e.g., tail probability of delay‐related measures) responds across the planning horizon. This figure therefore visualizes the interaction between reentrant demand amplification (feedback/retrial) and capacity decisions, and serves as evidence that the proposed procedure can steer the system performance toward the prescribed SLA target.

**Figure 4:**
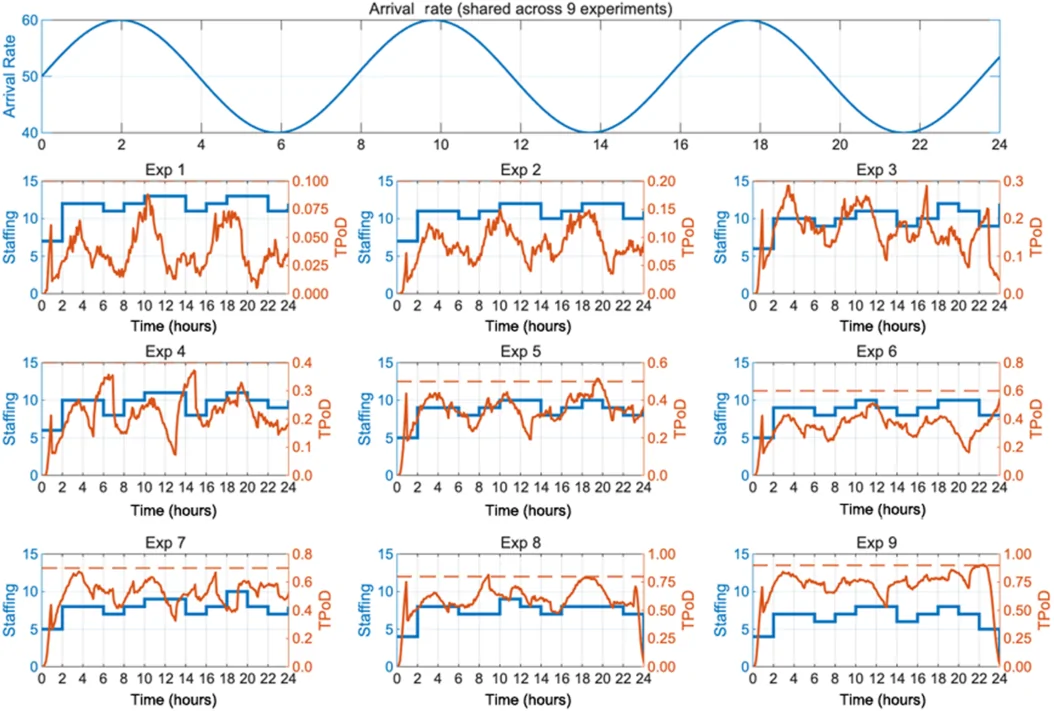
Performance evaluation of the dynamic physician rostering framework across nine experimental scenarios over a 24‐h cycle with post‐service feedback probability *p*_2_ = 0.7 and pre‐service retrial probability *p*_3_ = 0.1. The top panel shows the shared time‐varying patient arrival rate. Panels Exp 1–9 present the recommended staffing levels (step function, left axis) and the realized TPoD trajectories (solid curve, right axis). The red dashed lines indicate the predefined TPoD service level targets (*α*) for each experiment, confirming the algorithm's capability to maintain operational safety under fluctuating demand. Exps 1–9 respectively represent the TPoD ranging from 0.1 to 0.9. Exp, experiment; TPoD, tail probability of delay.

Additionally, as depicted in Figure 4, we comprehensively evaluate the performance of the proposed ISA across nine distinct experimental scenarios. The top panel illustrates the baseline patient arrival rate, which exhibits a strong, non‐stationary diurnal pattern over a 24‐h operational cycle. The subsequent 3 × 3 grid (Exp 1 to Exp 9) details the dynamic capacity adjustments and the resulting system performance under varying service level constraints. In each subplot, the step function (left axis) represents the recommended physician staffing level generated by the ISA. The fluctuating solid curve (right axis) tracks the realized TPoD, while the horizontal red dashed line denotes the prescribed TPoD safety threshold (*α*) specific to that experiment.

The results clearly demonstrate the robustness of the dynamic rostering framework. Despite severe demand fluctuations, the step‐wise capacity adjustments effectively track the arrival peaks, consistently constraining the realized operational risk (i.e., TPoD) below or tightly aligned with the targeted service level agreements across all tested parameter settings.

## 4 Discussion

This study offers critical insights for hospital administrators and ED directors aiming to enhance operational resilience and patient safety. We have a discussion about managerial insights and limitations in this section.

### 4.1 Managerial Insights

By shifting from static averages to dynamic flow modeling, our findings support three key management strategies:

(1) Seeing the “hidden” workload of returning patients.

Traditional capacity planning often just counts the patients walking in the door, completely missing the hidden workload created when frustrated patients leave and come back later (often sicker), or when they must re‐enter the queue after initial tests. For hospital managers, the takeaway is clear: investing in enough staff upfront to reduce initial wait times stops this vicious cycle. Getting it right the first time doesn't just improve the immediate patient experience; it actually reduces the total number of visits by preventing these avoidable “bounce‐backs”.

(2) Looking beyond “average waits” to protect patient safety “average wait time” is a deceptive metric.

A good average can easily hide the fact that a few vulnerable patients waited dangerously long periods during a sudden rush. Instead of managing by averages, scheduling should target a strict safety net, such as ensuring no more than 5% of patients wait longer than an hour. By staffing specifically to prevent these extreme delays (our tail probability of delay metric), hospital leaders can guarantee true clinical safety and reliably meet strict access standards, rather than just chasing a nice‐looking overall average.

(3) Replacing rigid schedules with smart, flexible shifts.

Setting a flat number of doctors for the whole day guarantees two things: severe bottlenecks during rush hours, and wasted idle capacity during quiet times. True lean scheduling means matching staffing exactly to the highest periods of operational risk. By deploying flexible “swing shifts” to reinforce the team right before wait‐time breaches are predicted to spike, managers can eliminate crowding without overspending on payroll. This not only keeps labor costs in check but also smooths out the chaotic workload peaks that rapidly burn out clinical staff.

### 4.2 Implementation Challenges in Practice

We explicitly discuss that a theoretical framework must be critically evaluated against real‐world hospital operations:

(1) Data requirements: Implementing our model requires high‐resolution tracking of endogenous patient flows. We noted that many current electronic health record systems struggle to accurately timestamp LWBS events or automatically link return visits to the initial abandonment. Upgrading data capture protocols is a necessary prerequisite.

(2) System integration: We discussed the technical challenge of integrating the ISA outputs with legacy hospital scheduling systems, suggesting the need for modular software interfaces.

(3) Staff resistance: Transitioning from predictable, static shifts to dynamic, demand‐responsive rostering may disrupt clinical staff routines and cause friction. We emphasized that successful implementation requires strong change management, ensuring that flexible shifts (e.g., swing shifts) are introduced gradually and involve staff input to balance operational efficiency with provider well‐being.

## 5 Conclusions

This study bridges the gap between theoretical patient flow models and the operational reality of EDs, where patient arrivals are rarely linear. By explicitly modeling the “churn” of patient re‐entries, both from those who leave without being seen and those requiring rework, we have demonstrated that neglecting these endogenous dynamics leads to significant mis‐staffing and persistent access blocks.

Our findings underscore that operational resilience cannot be achieved solely by adding resources, but by dynamically aligning physician capacity with the “risk profile” of patient wait times. The proposed framework confirms that targeting the TPoD offers a far more robust safety net than relying solely on traditional average‐based metrics. Furthermore, the success of the simulation‐based ISA approach illustrates that complex, reentrant loops can be effectively managed to balance clinical safety with cost‐efficiency.

From an implementation perspective, the proposed framework can be integrated into routine ED operations as a rolling planning tool: managers can update demand inputs (including estimated revisit/return rates) on a daily or weekly basis, generate an hour‐by‐hour staffing recommendation, and translate it into actionable shift templates (e.g., adding short “overlay” shifts during high‐risk windows). Importantly, by reducing avoidable congestion and repeat visits, such risk‐aware rostering can help smooth workload peaks and relieve unnecessary overtime pressure on frontline clinicians.

Despite the operational insights generated by our dynamic rostering framework, we must acknowledge a significant limitation of this study: the absence of empirical validation using real‐world clinical data. While our discrete‐event simulations provide a rigorously controlled environment to isolate and analyze the complex mechanics of endogenous patient flows (i.e., LWBS and subsequent clinical revisits), the lack of empirical testing limits the immediate generalizability of our quantitative results. The simulated parameters, though grounded in existing literature, may not fully capture the idiosyncratic constraints and behavioral nuances of diverse hospital environments. Therefore, future research must critically prioritize empirical validation.

Several directions merit further research to expand the applicability of this framework. Future work should consider clinical heterogeneity by incorporating multi‐class acuity‐dependent routing and shared downstream resources (e.g., imaging and labs). Additionally, validating these models with high‐resolution data from multi‐site hospital systems will further refine these managerial insights for broader implementation.

## Author Contributions

**Xu Dai:** methodology, writing – original draft. **Peng Gao:** supervision, writing – review and editing. **Xiaolei Xie:** supervision, writing – review and editing, funding acquisition.

## Ethics Statement

The authors have nothing to report.

## Consent

The authors have nothing to report.

## Conflicts of Interest

Xiaolei Xie is the Section Editor of *Health Care Science* Editorial Board. To minimize bias, he was excluded from all editorial decision‐making related to the acceptance of this article for publication. The remaining authors declare no conflict of interest.

## Supporting information


Supporting File 1

Supporting File 2

## Data Availability

Research data are not shared.
